# Shelter-building behavior and natural history of two pyralid caterpillars feeding on *Piper stipulaceum*

**DOI:** 10.1093/jis/14.1.39

**Published:** 2014-01-01

**Authors:** Mariana Abarca, Karina Boege, Alejandro Zaldívar-Riverón

**Affiliations:** 1 Departamento de Ecología Evolutiva, Instituto de Ecología, Universidad Nacional Autónoma de México (UNAM), México D.F., México; 2 Department of Biological Sciences, George Washington University, 2023 G St. NW Suite 340, Washington, D.C., USA; 3 Colección Nacional de Insectos, Instituto de Biología, Universidad Nacional Autónoma de México (UNAM), México, D.F., México

**Keywords:** Chrysauginae, herbivory, *Lepidomys*, Pyralidae, *Tosale,*, trenching, tropical dry forest

## Abstract

Shelter-building behavior by caterpillars provides a mechanism of defense against predators, microenvironment enhancement, and in some cases nutritional benefits. This study provides a detailed description of the life cycle and shelter-building process of caterpillars, and identifies constraints and factors influencing this adaptive behavior in *Lepidomys* n. sp. near *proclea* Druce (Pyralidae: Chrysauginae), a tropical dry forest pyralid. Five macroscopic larval instars were detected during the life cycle, and activities performed during shelter-building were categorized and timed. Caterpillar predators were identified, and 20% of all collected larvae died due to attack by parasitoid wasps. Shelter-building behavior was found to be constrained by the ontogenetic stage of caterpillars and influenced by leaf size of the host plant, *Piper stipulaceum* Opiz (Piperales: Piperaceae) *.* A similar pattern of shelter-building behavior exhibited by *Tosale* n. sp. near *cuprealis* larvae that coexisted in the same host plant is also described. Larvae of the second species were significantly less abundant than those of *Lepidomys* and hatched one month later in the rainy season, which could indicate some competitive interactions between these two pyralid species.

## Introduction

Shelter-building is a common behavior among the order Lepidoptera, and has been recorded for larvae of at least 24 families, which represents 25% of all families of Lepidoptera ( Lill and Marquis 2007 ). Lepidopteran shelters can be made out of silk, twigs, or leaves that are rolled, folded, or tied. Shelters can take different shapes, from a simple folded leaf fragment to complex silk tunnels and structures involving multiple leaves sewn together ( DeVries 1987 ; Stehr 1987 ; Scoble 1992 ; Huertas 2006 ). Caterpillars, unlike other shelter-building insects, lack specialized measurement structures like antennae, though many are capable of building regular shelters using their body size as a measurement scale ( Weiss et al. 2003 ; Hansell 2005 ). Caterpillars also follow a stereotyped silking pattern, building standardized shelters of the same size and shape according to the species and its developmental stage ( Lind et al. 2001 ; Darling 2003 ; Greeney and Jones 2003 ; Weiss et al. 2003 ). Ontogenetic restrictions in shelter-building behavior are mainly due to mandible size and strength, which determine the caterpillar’s ability to cut and fold foliage and also restrict its feeding habits ( Hochuli 2001 ; Greeney and Jones 2003 ; Weiss et al. 2003 ; Ide 2004 ). Therefore, shelter building is often restricted to the later larval instars ( Gaston et al. 1991 ).

Costs and benefits of shelter-building behaviors have been studied in several species of lepidopterans ( Lill and Marquis 2007 ; Marquis and Lill 2007 ). Benefits include light shielding ( Sandberg and Berenbaum 1989 ), microclimate enhancement ( Hunter and Willmer 1989 ; Larsson et al. 1997 ), protection from predators ( Damman 1987 ; Eubanks et al. 1997 ), and increase in plant quality ( Sandberg and Berenbaum 1989 ; Sagers 1992 ). In the present study, the shelters that were found provided defense against predators and, to a lesser extent, nutritional benefits, which change with shelter occupation time ( Abarca and Boege 2011 ). Potential costs of the shelter-building habit include the time and energy spent building them ( Fitzgerald et al. 1991 ; Berenbaum et al. 1993 ; Fitzgerald and Clark 1994 ) as well as increased risk of parasitoid attack relative to non-shelter-building species ( Marquis and Lill 2007 ). Shelters are associated with high parasitoid attack ( Lill 1999 ; Lill and Marquis 2007 ) because they protect caterpillars from predation, so they may represent a better choice for parasitoids to increase their survival ( Gentry and Dyer 2002 ). Shelters also concentrate visual and chemical cues, which can attract parasitoids ( Weiss et al. 2003 ).

Detailed descriptions of the shelter-building habit are available for various species such as the rice leaf folder, *Caloptilia serotinela* (Lepidoptera: Pyralidae) ( Fraenkel and Fallil 1981 ); the red admiral caterpillar, *Vanessa indica* Herbst (Lepidoptera: Nymphalidae) ( Ide 2004 ); and several skippers (Hesperiidae), such as the silver-spotted skipper, *Epargyreus clarus* Cramer (Lepidoptera: Hesperiidae) ( Weiss et al. 2003 ) and *Falga jeconia ombra* Evans (Lepidoptera: Hesperioidea) ( Greeney and Warren 2009 ). The diversity of shelters and the shelter-building techniques used for their construction are still largely underrepresented in the literature, and little is known regarding leaf selection and natural history constraints to shelter building, particularly in tropical systems. In this context, the aim of this study was to characterize the shelter-building behavior and life cycle of an undescribed species of pyralid larva, *Lepidomys* n. sp. near *proclea* Druce (Pyralidae: Chrysauginae) in a Mexican tropical dry forest, and the species’ association with parasitoidism rates. Caterpillars’ choice of leaves with which to build shelters and the ontogenetic constraints associated with this behavior were also assessed.

## Materials and Methods

### Study site and species

**Figure 1.**
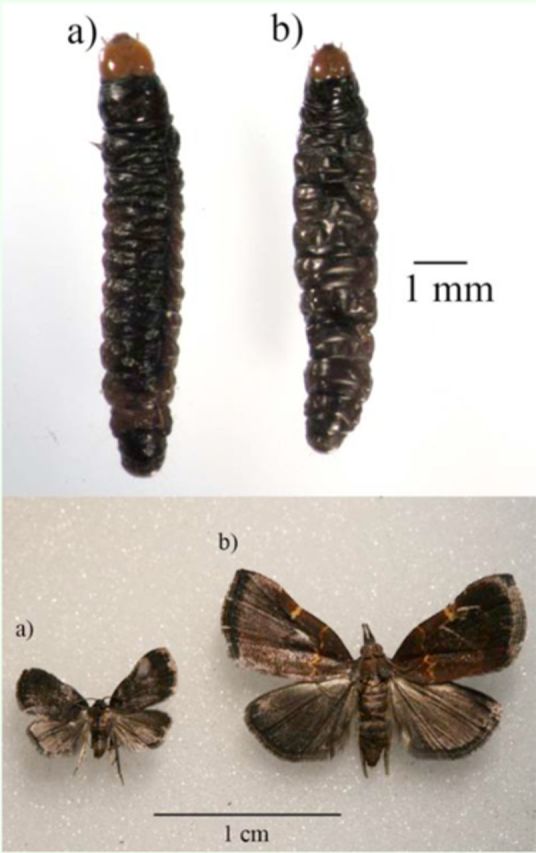
A. *Tosale* larvae and adults. B. *Lepidomys* larvae and adults *.* High quality figures are available online.

This study was carried out in 2008 at the Chamela-Cuixmala Biosphere Reserve, which is located on the Pacific coast of Jalisco, Mexico, between N 19°30′and W 105°3′( Noguera et al. 2002 ). The vegetation in this area is dominated by tropical dry forest ( Lott et al. 1987 ), with a mean annual precipitation of 788 mm and high variation between years. *Lepidomys* n. sp. near *proclea* Druce ( *Lepidomys* hereafter) coexisted with another undescribed pyralid, *Tosale* n. sp. near *cuprealis* Hampson (Lepidoptera: Pyraloidea: Pyralidae: Chrysauginae) ( *Tosale* hereafter). Due to the poor knowledge of these taxa (A. Solis, personal communication), their actual phylogenetic relationship is still unknown. Both species built identical shelters on the foliage of the shrub *Piper stipulaceum* Opiz (Piperales: Piperaceae) and fed inside them. Their larvae were indistinguishable in the field ( Figure 1 ), and they belong to the neglected pyralid subfamily Chrysauginae, which includes a number of non-monophyletic, monotypic genera (A. Solis, personal communication). Arthropod herbivores from the Chamela region are strongly seasonal, and their activity is determined by rainfall patterns, with peak activity at the beginning of the rainy season (July–November) and a restricted activity period for adults ( Pescador-Rubio et al. 2002 ).

### *Lepidomys* life cycle

To describe *Lepidomys* life cycle, larvae were collected from existing shelters on *P. stipulaceum* during the rainy season from July to October 2008. All larvae were reared in plastic containers and fed with fresh *P. stipulaceum* foliage until caterpillar death or pupation. A randomly-selected subsample of pupae was weighed using a digital balance (Acculab Sartorius, www.acculab.balances.com ). Emerged adults were identified and maintained in captivity for breeding and fed with sugared water. The total number of instars was determined from direct measurements of head capsule width in early instars and from moulting events in later instars. In the case of the early instars, recently-hatched *Lepidomys* caterpillars were monitored, and subsamples were subsequently sacrificed as they developed in order to obtain measurements of their head capsules. All caterpillar samples were preserved in 70% alcohol and photographed with a PowerShot 620 digital camera (Canon, www.canon.com ) adapted to a Discovery V.8 (Zeiss, www.corporate.zeiss.com ) stereoscopic microscope. The frequency distribution of the head capsule width was analyzed to estimate the number of instars of these samples. Since the frequency distribution was found to be bimodal, head capsule measurements were separated into two groups, with the k-means clustering method using MATLAB 7.0 (MathWorks, www.mathworks.com ), and differences between groups were assessed using a Wilcoxon test. The number of instars during further development was directly assessed from observed moulting events and from head capsule measurements of moults using the previously-described set up.

### Shelter-building behavior

A total of 31 collected caterpillars, presumably belonging to the last three instars of *Lepidomys* , were observed in the field to describe the shelter-building process. To avoid including *Tosale* individuals in the sample, caterpillars were reared in captivity after the building process, and only those that successfully emerged and were identified as *Lepidomys* were included in the description. Each caterpillar was placed on a leaf of *P. stipulaceum* , and up to four larvae were simultaneously observed. The caterpillars’ activities and positions were recorded continuously, as were any displays of behavior (see results for a list and description of the behaviors characterized). Because caterpillars move around the plant before selecting a leaf to build their shelter, each larva was placed in a different plant to avoid overlapping. Observations lasted until the shelter was finished (up to six hours), which occurred when the shelter completely concealed the caterpillar inside it.

Since leaf size can influence a caterpillar’s choice when selecting a leaf to build a shelter, measurements of leaf length and width as well as petiole width of all recently-built leaf shelters were taken and compared to a systematically-selected sample of available leaves. Because leaf length and width are positively correlated in *P. stipulaceum* ( *r*^2^ = 0.783, *P* < 0.0001), only width was reported in the results. Measurements of the available leaves were taken monthly from July to October. Every month, 40 leaves from each of 20 plants were systematically selected so they were evenly spaced across the plant regardless of leaf size. Comparisons among months were made to record leaf size change through time. As leaf and petiole measurements did not fit a normal distribution, comparisons of leaf size among months were performed with a Fried-man’s test using the program STATISTICA (StatSoft, www.statsoft.com ).

To compare the dimensions of leaves selected by caterpillars for shelter-building with the dimensions of leaves available in the plant, measurements of the 133 shelters that were collected across the season were compared to a subsample of 133 available leaves. This subsample was randomly chosen from the database of measured leaves (800 by month). Because average leaf size differed among months, comparisons were made using separate Wilcoxon tests for July and September using JMP software (SAS, www.jmp.com ).

### Parasitoid association

Because parasitized larvae could not be easily identified, sequences of the DNA barcoding locus were obtained (650 bp of the cytochrome oxidase I mitochondrial DNA gene; Hebert et al. 2003 ) to distinguish *Lepidomys* from *Tosale* dead larvae. All remains of parasitized larvae (head capsule and fragments of epidermis) and emerged adult wasps were collected, preserved in 70% ethanol, and stored at room temperature. Barcode sequences for adults of the two moth species were also generated for comparison with the sequences obtained from larval remains in order to confirm the adults’ identities.

Non-destructive genomic DNA extractions were carried out for adult wasps and lepidopteran larvae remains with the DNAeasy extraction kit (Qiagen, www.quiagen.com ), leaving the whole individuals digesting overnight in 100 µL of Qiagen ATL buffer and 20 µL of proteinase K. DNA extractions for adult moths were carried out with the above kit using a single leg. All amplifications were carried out using the LepF1/LepR1 primers ( Hebert et al. 2004 ) (LEP-F1: 5'-ATT CAA CCA ATC ATA AAG ATA T-3'; LEP-R1: 5'-TAA ACT TCT GGA TGT CCA AAA A-3'). PCRs were carried out in a 25 µL total volume using 2.5 µL of 10x PCR buffer, 1 µL of MgCl2, 0.25 mM of each dNTP, 0.4 µM of each primer, 0.2 µL of platinum Taq polymerase (Life Technologies, www.lifetechnologies.com ), 5 µL of DNA template, and 12 µL of ddH2O. The PCR program had an initial 1 min denaturation at 94°C, followed by 5 cycles at 94°C for 30 sec, 48°C for 40 sec, and 72°C for 1 min, as well as 30–35 cycles at 94°C for 30 sec, 54°C for 40 sec, and 72°C for 1 min. A 10 min extension period of 72°C followed the final cycle. PCR products were purified using the Millipore clean-up system ( www.millipore.com ). Sequences were edited with Sequencher version 4.0.5 (Gene Codes, www.genecodes.com ) and aligned manually based on their translated amino acids. All the COI obtained were deposited in GenBank. Uncorrected genetic distances trees were obtained with PAUP version 4.0b10 (Swofford 2002) using all the generated lepidopteran DNA sequences. All adult wasps were identified to genus level using relevant literature, and their barcode sequences were compared with those deposited in GenBank using the basic local alignment search tool (BLAST).

## Results

### Larval collections

A total of 165 larvae were collected in the field, of which 45% belonged to *Lepidomys* and 28% to *Tosale* . The remaining larvae (27%) died before pupation and could not be identified. Larvae of *Lepidomys* and *Tosale* were first observed in July and August, respectively. Both species are very similar at their immature stages but easily distinguishable as pupae and adults due to differences in size: *Lepidomys* pupae weighed almost six times more than *Tosale* pupae ( *Lepidomys* : 41 ± 1 mg, n = 62; *Tosale* : 7.7 ± 0.3 mg, n = 20, mean ± SEM; Figure 1 ). Both species are multivoltine and are present on *P. stipulaceum* foliage until the end of the rainy season. Three generations of *Lepidomys* were observed from July to October. Only *Lepidomys* was sufficiently abundant to establish a colony in captivity. Nevertheless, from observations of the progeny of one mated *Tosale* couple it could be determined that *Tosale* had two larval stages that were distinguishable by larval size and head capsule color (black and reddish head larvae). Observations also revealed that both *Lepydomis* and *Tosale* larvae built and occupied shelters throughout their entire development.

### *Lepidomys* life cycle

**Figure 2.**
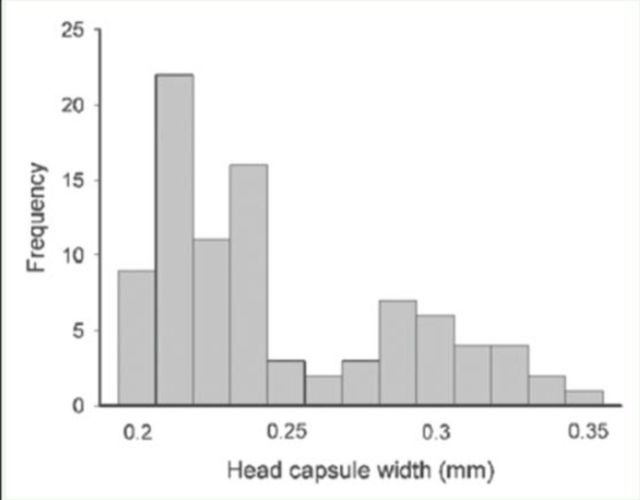
Frequency distribution of head capsule width measurements of the two black head instars of *Lepidomys* . High quality figures are available online.

Eggs and neonates are microscopic, so their behavior and morphology could not be fully described. The subsequent five macroscopic instars lasted a total of 30 days in captivity when fed a fresh foliage diet, although larval development in the field can be affected by food quality and thus can take longer ( Abarca and Boege 2011 ). The first two macroscopic instars had black head capsules and were gregarious. These two stages were identified from a bimodal distribution of the head capsule measurements ( Figure 2 ), and it was assumed that each group corresponded to a different instar. The first group included 61 larvae whose head capsule ranged from 0.2– 0.26 mm, with a mode of 0.22 mm. The second group, with 29 individuals, had head capsules ranging from 0.27–0.35 mm with a mode of 0.29 mm. Measurements of the two groups were significantly different (χ1 ^2^ = 59.7, *P* < 0.0001). These two black head instars lasted around seven days in captivity.

The next three instars had reddish head capsules and were determined from moulting observations. The 3rd and 4th macroscopic instars were difficult to distinguish based on head capsule size. Their measurements ranged from 0.4 to 1.4 mm, but they overlapped. For example, one individual had a head capsule of 0.77 mm in the 3rd instar and 0.88 mm in the 4th, so the difference was only 0.11 mm. The last instar was easily distinguishable from the rest, with an average head capsule width of 1.45 ± 0.02 mm (mean ± SEM, n = 54). Together, these three reddish head instars lasted from 19 to 23 days. When the last-instar larvae were ready to pupate they left their shelters and dropped to the soil, where they built a soil and silk cocoon. Adults emerged after 12.4 ± 0.21 days at dusk (mean ± SEM, n = 37) and lived up to 10 days in captivity. All mating events observed were performed before dawn and individuals remained coupled for periods longer than one hour.

### Shelter-building behavior

**Figure 3.**
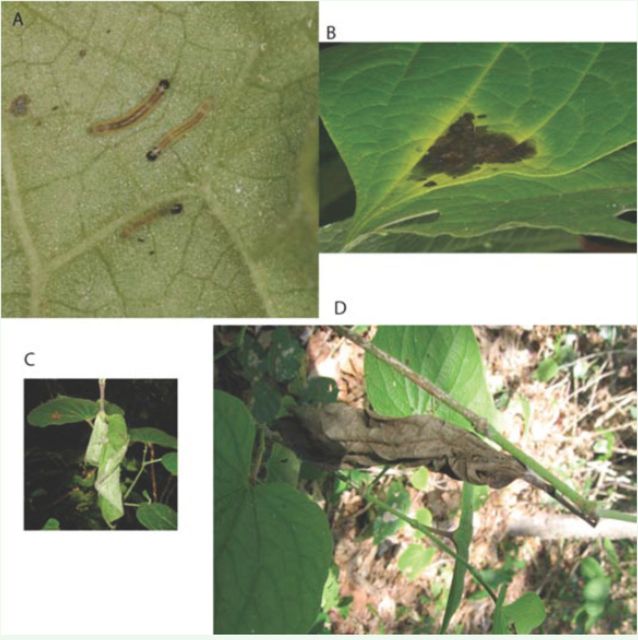
Leaf structures inhabited by *Lepidomys* according to their ontogenetic stage: A. Black head individuals. B. Leaf tie. C. Trenched leaf shelter. D. Nest. High quality figures are available online.

*Lepidomys* larvae built two types of shelters over their lifetime: leaf ties and trenched complex shelters. Eggs were laid in masses in the space where two leaves overlapped. The small black head instars ( Figure 3A ) silked overlapping leaves together and inhabited resulting leaf ties. These ties turned a yellowish color ( Figure 3B ) as a consequence of larval activity, and were shed from the plant after several days of larval occupancy. Predators such as spiders and scorpions were observed using these leaf ties as shelters.

**Figure 4.**
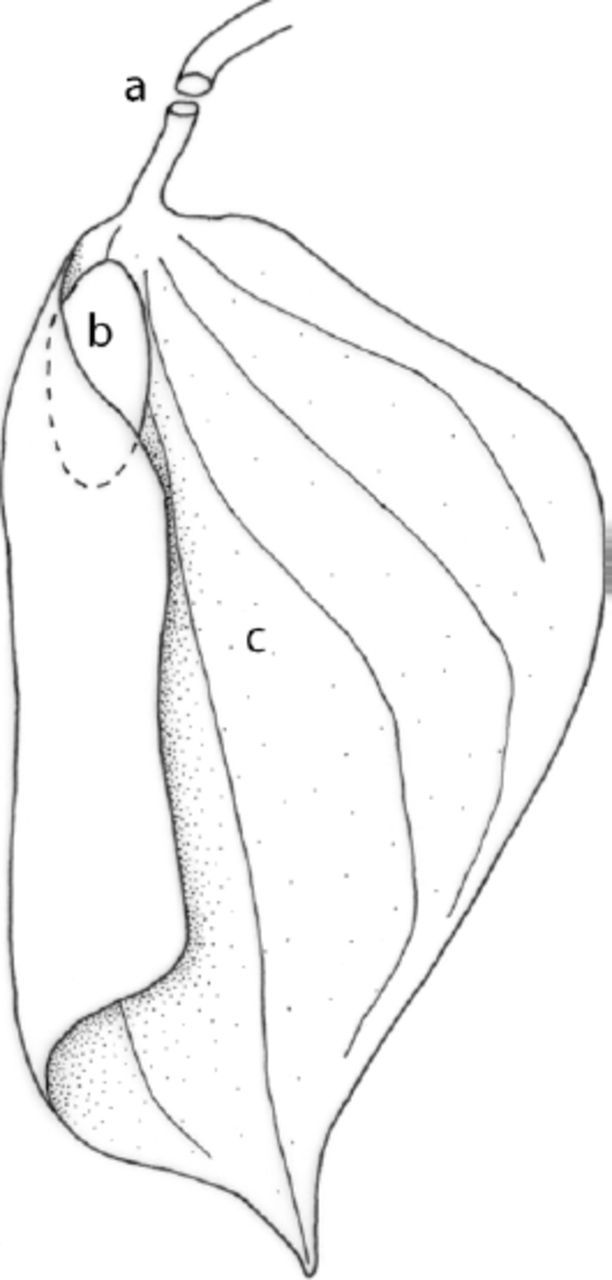
Diagram of an opened shelter A. Trench. B. Main chamber. C. Opened secondary chamber. In a natural shelter the right part of the leaf would fall down and to the left, covering the main chamber and the secondary chamber. High quality figures are available online.

When caterpillars reached the first reddish head instar, they became solitary and built complex trenched leaf shelters ( Figures 3C , 4 ). These shelters were composed of a trench, a small main chamber only large enough for the caterpillar to fit inside, and a larger secondary chamber that was often colonized by other arthropods ( Figure 4C ). On rare occasions, two or three caterpillars were found in the same leaf shelter, each in an individual main chamber. When two caterpillars encountered each other during leaf shelter construction, they reacted aggressively. They hit each other with their heads and the resident caterpillar kept the leaf. Early-instar *Lepidomys* individuals could be occasionally found in “nests” ( Figure 3D ), which are dry shelters abandoned by older caterpillars and subsequently used as oviposition sites by females. *Tosale* larvae occupied most of these nests.

**Figure 7.**
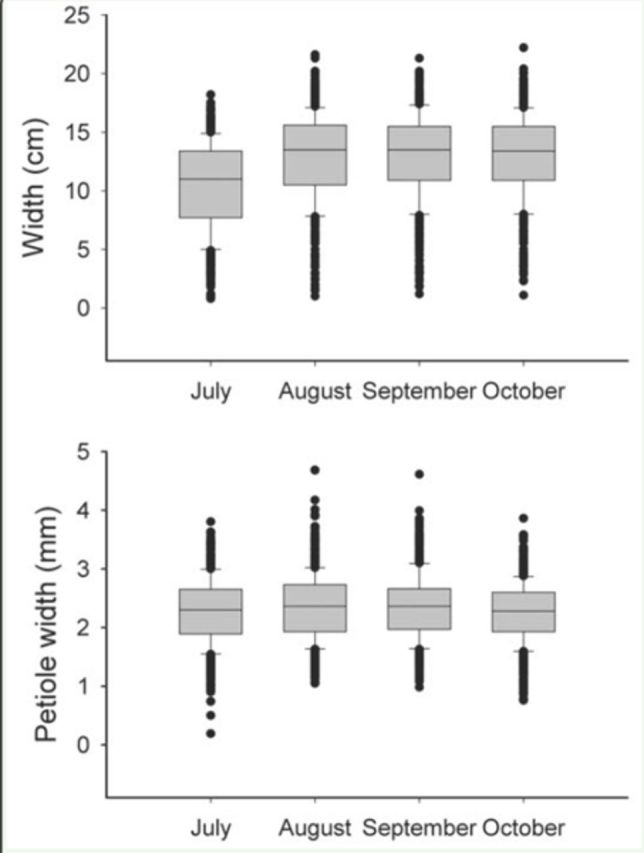
Width and petiole width of available leaves of *Piper stipulaceum* in Chamela, Jalisco over time in 2008. High quality figures are available online.

Activities that *Lepidomys* larvae performed during shelter building were categorized as follows. First, caterpillars moved around the leaf before they began the building process. Most caterpillars (14 out of 21) visited at least two leaves before starting the trenching process, but some visited as many as seven leaves before choosing their preferred leaf. Caterpillars chose leaves that were 12 cm long and wide in both July and September. During July, selected leaves were significantly larger than the average available leaves (width: *χ* 1 ^2^ = 6.71, *P* = 0.0096, n = 108; petiole width: *χ* 1 ^2^ = 8.56, *P* = 0.0034, n = 108). In September, selected leaves did not differ from those available (width: *χ* 1 ^2^ = 0.59, *P* = 0.44, n = 148; petiole width: *χ* 1 ^2^ = 0.029, *P* = 0.87, n = 148). Available leaves were smaller in July than in the rest of the season (width: *χ* 3 ^2^ = 278.4, *P* < 0.0001, n = 3084; petiole width: *χ* 3 ^2^ = 21.2, *P* = 0.0001, n = 3083), as shown in Figure 7 ; however, the size of the leaves chosen to build shelters did not differ significantly between July and September (width: *F* = 0.69, *P* = 0.41, n = 132; petiole width: *F* = 2.71, *P* = 0.10, n = 132).

**Figure 5.**
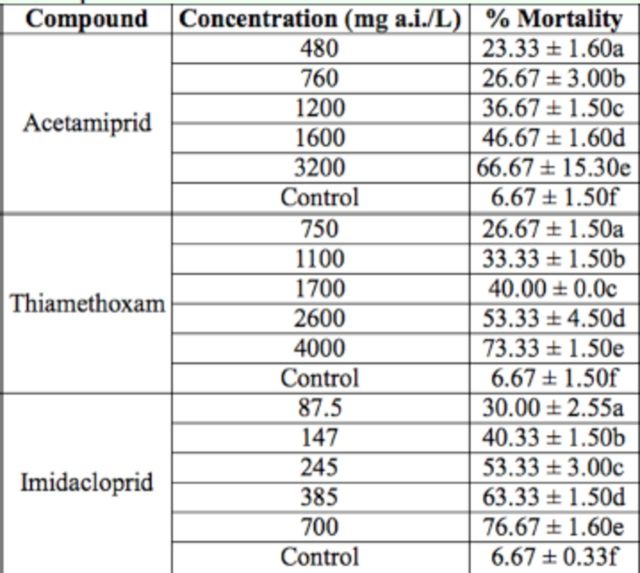
Steps of the shelter-building process: A. Petiole trench. B. Silking of the trenched site. C. Silking of the next node. D. Leaf silking. E. Folded leaf. F. Fresh shelter capsule. G. Open dry shelter capsule with a last instar larva. H. Fresh shelter. I. Dry shelter. J. Attachment of a new leaf to the dried shelter, where (1) shows the original shelter, (2) the new feeding site, and (3) the trenched petiole of the new leaf. High quality figures are available online.

Once the caterpillar had chosen a leaf, it se-cured itself with a silk strand to the leaf base and trenched the petiole ( Figure 5A ). Caterpillars ate the tissue they removed during this process. In general, trenching was finished when only the inferior part of the epidermis remained. Then the trenched region was silked ( Figure 5B ), securing the leaf to the plant. Although it was rare for a caterpillar to keep trenching until the leaf fell from the plant, it was occasionally observed in the present study. Sometimes caterpillars silked beyond the trenching site, in the closest node, securing the whole petiole to the plant ( Figure 5C ). After petiole silking, caterpillars went to the center of the leaf base ( Figure 5D ) and extended silk strands perpendicular to the petiole, folding the leaf ( Figure 5E ) until both sides of the leaf touched. During this process it is common for caterpillars to eat portions of the leaf base tissue, which probably facilitates folding. To build the main chamber, the caterpillar went to one of the leaf ends, rolled it and silked two portions of the leaf together, and subsequently cut the edges ( Figure 5F , G ). Caterpillars spent most of their resting time inside the main chamber and they left it only to feed within the secondary chamber and reinforce the shelter’s silk joints. The main chamber was covered by the secondary chamber, which was often colonized by insects and spiders. Recently-built shelters ( Figure 5H ) remained occupied even when they were completely dry ( Figure 5I ) because caterpillars are able to feed on dry foliage ( Abarca and Boege 2011 ). Occasionally, caterpillars were observed trenching a fresh leaf and silking it to their shelter to feed on it without leaving the original shelter ( Figure 5J ).

**Figure 6.**
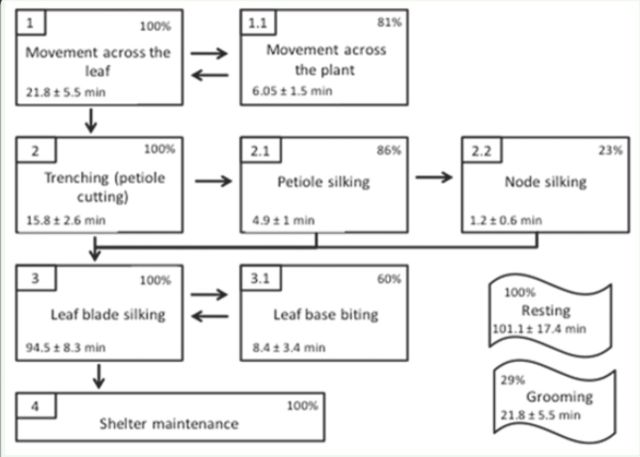
Diagram showing shelter-building activities. Each polygon shows an activity, the percentage of larvae that performed it, and its mean duration ± standard error (n = 21). Grooming and resting are not linked by arrows because they can occur at any time and interrupt any of the other behaviors. High quality figures are available online.

The sequence of activities and the time spent performing each of them during the shelter-building process is shown in Figure 6 . The whole process may last from three to six hours. The duration of step number four ( Figure 6 ), shelter maintenance, is not specified because it includes the rest of the occupation period, which can last up to two weeks. The building process was interrupted by periods of rest; all of these periods were grouped in the resting category. Resting and grooming may occur at any time and interrupt any of the other behaviors. The term grooming is used to designate the caterpillars’ action of rubbing their body with their mandibles. Caterpillars may return to the petiole to attach more silk days later if the shelter is unstable. During shelter building, caterpillars spent, on average, 78% of the time on the adaxial face of the leaf (mean ± SEM, 203 min ± 16), 10% on the petiole (32 min ± 5), 8% on the abaxial face (15 min ± 7), and 3% moving on the stems (7 min ± 2). On average, they spent 170.2 ± 15.8 min (n = 20) exposed on the leaf surface before the leaf folded and partially covered them.

### Natural enemies

More than nine predation events on larvae of either *Lepidomys* or *Tosale* were witnessed during shelter-building and other field observations. Recorded predators included spiders in the family Salticidae; carnivorous wasps, which attack larvae during the shelter building process; and coleopteran larvae, which are able to consume caterpillars inside their shelters. No wasps or birds were observed opening the shelters.

Table 1 shows the fate of all collected caterpillars by month. Twenty percent of all collected caterpillars died due to parasitoid attacks. Parasitoid wasps of the family Braconidae and the hyperparasitoid family Perilampidae were reared from *Lepidomys* remains. The host associations, which were confirmed by comparing DNA barcodes of parasitized larvae with those of adults of the two lepidopteran species, are listed in Table 2 . No parasitism events were recorded for *Tosale* individuals. Some of the larvae collected at the black head stage were parasitized, so it is likely that the parasitic attacks occur when the larvae are small and inhabit leaf ties.

**Table 1.**
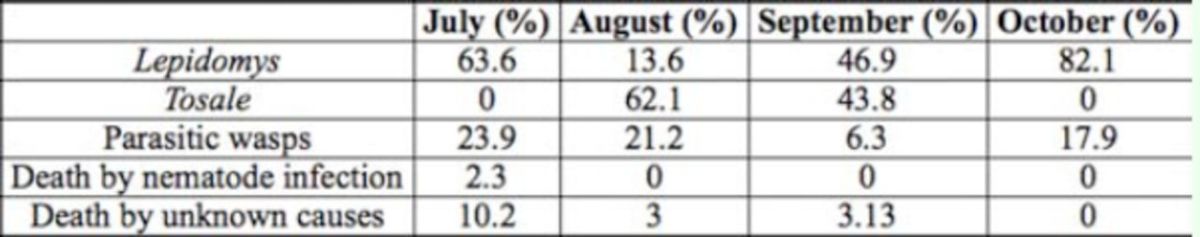
Seasonal abundance of *Lepidomys, Tosale* and their parasitoid wasps. Percentages indicate the proportion of all collected larvae that fully developed to the adult stage ( *Lepidomys* and *Tosale* ) or died before pupation

**Table 2.**
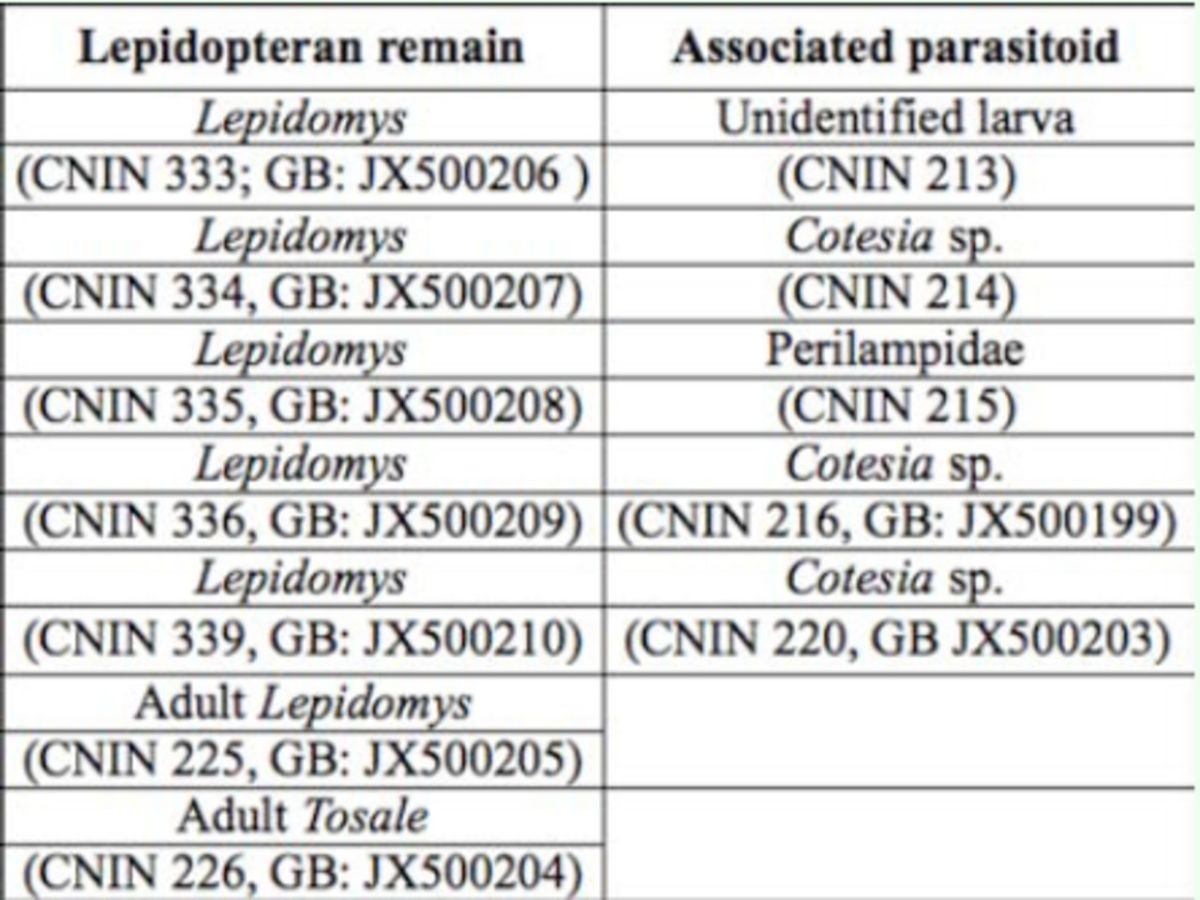
Lepidopteran remains and the corresponding associated parasitoid are shown. CNIN indicates the number assigned in the collection of the Instituto de Biología UNAM, GB indicates gen bank number. Barcode sequences of three other *Cotesia* sp individuals whose host could not be determined were also sequenced: CNIN 217: GB JX500200; CNIN 218:GB JX500201 andCNIN 219:GB JX500202.

## Discussion

It is common for shelter-building microlepidopterans to spend the whole larval stage protected, and some act as leaf or stem miners when they are too small to build a shelter ( Gaston et al. 1991 ). *Lepidomys,* however, spends the whole larval stage protected without changing its feeding habits because females lay eggs in leaf joints that are subsequently bound with silk strands by recently-hatched larvae or in previously-built shelters. Oviposition in leaf joints has also been reported for oak shelter-building moths ( Lill and Marquis 2004 ; Lill et al. 2007 ). Other microlepidopterans also oviposit in or near pre-existing leaf shelters ( Carroll and Kearby 1978 ; Carroll et al. 1979 ), where larval survival is higher compared to non-shelter leaves ( Cappuccino 1993 ). The only time period in which *Lepidomys* larvae are exposed on the leaf surface is during leaf selection and shelter-building. Leaf selection can take longer early in the season, when the foliage is still expanding, given the reduced availability of larger leaves; later in the season, caterpillars seem to have more large leaves to choose from because most of the leaves are already fully-expanded. The most time-consuming activities observed during shelter building were leaf silking and resting. Leaf silking was probably the most dangerous activity because it involved movement that further enhanced caterpillar conspicuousness (black larva moving on a green background). The developmental stage in which *Lepidomys* spent the dry season could not be confirmed. It is likely that pupae diapause during this time because the soil provides relatively constant temperature and humidity ( Denlinger 1986 ) and because most lepidopteran species spend the dry season as pupae in this and other tropical dry forests ( Janzen 1993 ; K. Boege, personal observation).

The shelter’s main chamber, where *Lepidomys* larvae spend most of their life cycle, is a simple structure that can be built without trenching the petiole ( Lind et al. 2001 ; Greeney and Jones 2003 ). However, caterpillars trench the petiole in spite of the predation cost involved, suggesting that it confers additional benefits. Trenching is likely to facilitate the rest of the shelter-building process because it provokes a turgidity loss that facilitates leaf folding ( Ide 2004 ; Hansell 2005 ). It may also have a nutritional function, since *Lepidomys* caterpillars feeding inside their shelters have been observed to have higher pupal mass than caterpillars feeding on non-trenched tissue ( Abarca and Boege 2011 ). Trenching usually prevents induction and flux of resin and latex ( Dussourd and Denno 1991 ; Chambers et al. 2007 ), which could be its function in this system. *Piper stipulaceum* plants do not produce latex or sap, but they could have alkaloid induction in response to herbivory damage, as reported for other species in the same genus ( Dodson et al. 2000 ). In addition, observations of leaf ties suggest that trenching is likely to prevent nutrient export from the leaf to the plant. Leaves bearing black head larvae showed signs of nutrient export (change to a yellowish color), and after a few days of occupation they were abscised from the plant. Herbivores can promote premature leaf senescence, which is manifested through color change from dark green, to yellow, to brown, and occurs when nitrogen is exported and finally leads to leaf abscission ( Blundell and Peart 2000 ; Morath et al. 2006 ). Premature leaf abscission has the potential to be an important larval mortality source ( Faeth et al. 1981 ), which could be the case for *P. stipulaceum* . Petiole trenching and silking may therefore be a strategy to ensure that nutrients stay in the leaf while it is still attached to the plant.

Conspecific agonistic interactions regarding shelters have been documented for few lepidopteran species. Parsnip webworms, *Depressaria pastinacella* Duponchel, fight over shelters by hitting one another other with their head capsules ( Green et al. 1997 ), which is similar to the interaction observed in the current study when two *Lepidomys* individuals found each other during shelter building. Other caterpillars compete over shelters using acoustic signals instead of actual physical contact, such as the common hook-tip moth, *Drepana arcuata* Walker, in which the intruder generally tends to lose ( Yack et al. 2001 ), and the cherry leaf roller, *Caloptilia serotinella* Ely, in which the resident is usually the one that elicits the more intense and frequent acoustic signals ( Fletcher et al. 2006 ). In the case of *Lepidomys* , only one agonistic interaction was observed, and the resident caterpillar kept the leaf. More observations would be necessary to characterize and document the prevalence of the aggressive behavior in this species.

The 20% parasitoid attack rate found for *Lepidomys* is high in comparison to other systems. For instance, Janzen and Gauld (1997) found parasitism rates that ranged from 1 to 6% in sphingids and saturnids of Ganacaste, Costa Rica. Parasitism rates found in the current study were closer to the 31% reported by Gentry and Dyer (2002) in a tropical rain forest. However, parasitism rates can vary significantly over time, as shown by Lill and Marquis (2001) in a temperate region. They found that parasitism levels suffered by the oak leaf-tying moth, *Psilocorsis quercicella* Clemens, another shelter builder, varied between 6.7 and 21.8% over the years of their study. All parasitized larval remains belonged to *Lepidomys* specimens, which started their larval activities in July with the first rains. If parasitoid populations are more abundant at the beginning of the rainy season, as is the case for Guanacaste, Costa Rica ( Hopkins and Memmott 2003 ), *Tosale* , which appeared a month later than *Lepidomys* , might be escaping parasitoids. However, because *Tosale* was less abundant than *Lepidomys* , the lack of observed parasitoidism events on this species could be a result of the small sample size. The most common parasitoid found in *Lepidomys* was the braconid *Cotesia* sp., but a number of specimens belonging to a Perilampidae hyperparasitoid were also reared. Perilampidae are known to be hyperparasitoids, attacking parasitoid flies (Tachinidae: Diptera) and wasps (Ichneumonoidea: Hymenoptera) ( Goulet and Huber 1993 ) that in turn attack lepidopteran larvae. In this case, they appear to have parasitized *Cotesia* pupae. Some caterpillars excrete frass or throw it outside the shelter ( Caveney et al. 1998 ), a behavior thought to minimize the concentration of chemical signals followed by parasitoids ( Weiss et al. 2003 ). *Lepidomys* larvae did not perform any of these behaviors in the current study, and their shelters concentrated frass in the secondary chamber.

In addition to serving as a refuge from predators, shelters function as microenvironment enhancers ( Hunter and Willmer 1989 ; Larsson et al. 1997 ). In the national park of Santa Rosa, Costa Rica, only 37% of studied lepidopterans feed while exposed on the surface of plants, and the rest are leaf miners or shelter builders ( Janzen 1988 ). It is therefore possible that the biotic and environmental conditions of this dry forest, with very similar conditions to the Chamela tropical dry forest, exert selective pressure on the caterpillars that favor concealment.

Shelter structure has been proposed as a behavioral trait that could be used to determine phylogenetic relationships because different lepidopteran families build characteristic shelters ( Greeney and Jones 2003 ). *Lepidomys* and *Tosale* build shelters different from those of other pyralid species, such as the rice leaf folder, *Cnaphalocrocis medinalis* Guenée, described by Fraenkel and Fallil (1981) , which silks and spins rice leaves to form rolls. Other shelter-building pyralids feed on plants that significantly differ from *P. stipulaceum* , such as grasses, maple trees, and pines ( Lill and Marquis 2007 ), and therefore their shelters are bound to differ. However, some similarities in the building process may occur within pyralid species, for example, the stitching behavior of *C. medinalis* is similar to *Lepidomys* ’ process of silking the base of the leaf because both involve repetitive movements to add silk strands ( Fraenkel and Fallil 1981 ). Detailed descriptions of the shelter-building process of a larger number of lepidopterans is needed to determine if there are specific and conserved traits across families.

Future studies involving *Lepidomys* or *Tosale* face the challenge of distinguishing between them. Working with lab colonies is a way to deal with this challenge by rearing the caterpillars until pupation, because *Lepidomys* pupae are significantly larger than those of *Tosale* . When rearing caterpillars is not possible or working with them involves high larval mortality, DNA barcoding techniques can be useful to identify larval remains. To increase the likelihood of collecting only *Lepidomys* individuals, sample collection can be carried out early in the season; when working with *Tosale* it would be better to start samplings later in the season.

Overall, the current study provides a detailed description of the life cycle and shelter-building behavior of *Lepidomys* larvae. Observations revealed ontogenetic constraints on this behavior such that only larvae in the latter instars were able to build trenched shelters, and morphological constraints due to the preference of larvae for larger leaves to build their shelters. *Lepidomys* and *Tosale* share a very similar larval appearance and identical shelter-building habits and coexist in the same host plant for most of the rainy season. Due to the poor knowledge of the phylogenetic relationships of these species, it is is unclear if they are so similar due to a shared evolutionary history or convergence. The likely competitive relationship between these species is yet to be determined and offers new and exciting avenues of future research.
